# Compact Bandwidth Enhanced Cavity-Backed Magneto-Electric Dipole Antenna with Outer Γ-Shaped Probe for GNSS Bands

**DOI:** 10.3390/s21113599

**Published:** 2021-05-21

**Authors:** Alexandre Causse, Kevin Rodriguez, Loïc Bernard, Ala Sharaiha, Sylvain Collardey

**Affiliations:** 1 IETR, University of Rennes 1, 263 Avenue du Général Leclerc, 35700 Rennes, France; ala.sharaiha@univ-rennes1.fr (A.S.); Sylvain.Collardey@univ-rennes1.fr (S.C.); 2 French-German Research Institute of Saint-Louis, 5 rue du Général Cassagnou, 68301 Saint-Louis, France; kevin.rodriguez@isl.eu (K.R.); loic.bernard@isl.eu (L.B.)

**Keywords:** magneto-electric, miniature, cavity-backed antenna, GNSS, wideband

## Abstract

In this paper, a wideband small cavity-backed magneto-electric (ME) antenna is proposed. This antenna is linearly polarized and designed to cover all the Global Navigation Satellite System (GNSS) bands. It exhibits small external dimensions of 90 × 90 × 40 mm^3^ (0.34 × 0.34 × 0.15 λ^3^ at lowest frequency) and achieves a wide impedance bandwidth of 40.5% (from 1.14 to 1.72 GHz) due to the excitation of a third resonance of the ME structure. It also provides a regular broadside gain of 5.2 dBi and stable radiation pattern in both E and H planes of the antenna.

## 1. Introduction

With the future introduction of modern GPS antennas and GALLILEO, several more frequencies and signals will be available that will improve the present high accuracy GPS capabilities. Hence, future GNSS (Global Navigation Satellite System) antennas will need to receive L band signals within a large band between 1.16 and 1.61 GHz. For flying platforms (e.g., medium and high altitude UVAs) evolving in multi-path free environments, the reception of GNSS signals (which are circularly polarized in order not to suffer from the ionosphere effects) can be efficiently performed with a linearly polarized (LP) antenna, as it is also in [1,2] for handheld devices. Indeed, the levels of the signals transmitted to the GNSS receiver are then 3 dB weaker than with a circularly polarized antenna, but this application case presents lower constraints on reception level due to the absence of reflections for the incoming signals. To equip such platforms, a low profile and cavity-backed linearly polarized antenna covering the frequency range from 1.16 to 1.61 GHz is required (the cavity brings some mechanical robustness to the antenna and facilitates its integration to the carrier).

Magnetoelectric (ME) dipole antennas have been widely studied since its introduction by K. M. Luk in 2006 [3]; by combining and exciting a magnetic dipole and an electric dipole together, the complementary antennas show good electrical characteristics, including wide impedance bandwidth (BW) of 47%, stable gain of about 8 dBi, low back radiation, low cross-polarization, and symmetrical E- and H- plane radiation patterns. In the last decade, several improved designs have been presented where efforts were made to increase the BW by modifying the shape of the dipoles [4], using parasitic elements [5] or specific cavities [6] and defected ground structures (DGS) [7]. However, these designs have an obvious drawback that the large antenna height is about one-quarter wavelengths, which is inappropriate to some practical applications. Moreover, these modifications lead to antennas having a wider BW but with an important increase in its electrical dimensions, especially for the ground plane. Other works to obtain low-profile ME dipoles have been made, by bending the magnetic dipole [8,9] or using metamaterial [10], which leads to a maximum height reduction of 61% and an impedance BW of only 28%. All these structures need a ground plane size of approximately λ at the center frequency.

Cavity antennas were investigated for decades and for a large range of applications such as mobile communications, telemetry of satellite communications, among others. F. Zavosh and J.T Aberle proposed in 1996 [11] a cavity-backed stacked patch antenna, with an increased BW, in comparison to conventional microstrip antennas. More recently, a resonant cavity antenna was proposed [12] using a non-uniform partially reflecting surface. This method enables a wideband 3 dB gain bandwidth of 22% for the antenna but implies a large ground plane radius of 1.1 λ_c_. Miniaturized cavity antennas [13,14] were also investigated. These antennas provide a single resonance resulting in small ground plane sizes and narrowband antennas. This type of antenna preserves the advantage of low profile and high front to back ratio but is not wideband enough for the application targeted in this paper.

Cavity-backed magneto electric antennas have also been developed for some specific applications such as communication systems, outdoor applications, satellite communications, or radar applications [6,15,16,17], providing equivalent impedance BW and good radiation properties with cavity sizes of approximately λ at center frequency. Reducing the cavity size drastically affects the BW of the antenna.

In this paper, a novel and simple wideband magneto-electric dipole antenna with a small dielectric loaded cavity backed is proposed. This multi-resonant structure has a large BW up to 40.6% while maintaining a low profile and a small cavity dimension less than 0.45λ_c_. This behavior is obtained by optimizing the Γ-shaped probe position as well as its coupling with the electric dipole, covering the frequency range from 1.14 to 1.72 GHz. The final antenna has smaller dimensions than the state of the art while keeping wideband properties, as demonstrated at the end of the article. The paper is organized as follows: Section 2 focuses on the excitation of the structure and on the originality of the proposed solution, Section 3 covers the design methodology, whereas Section 4 presents the simulation and experimental results and Section 5 concludes the paper.

## 2. Outer Probe Excitation for BW Enhancement

**Figure 1:**
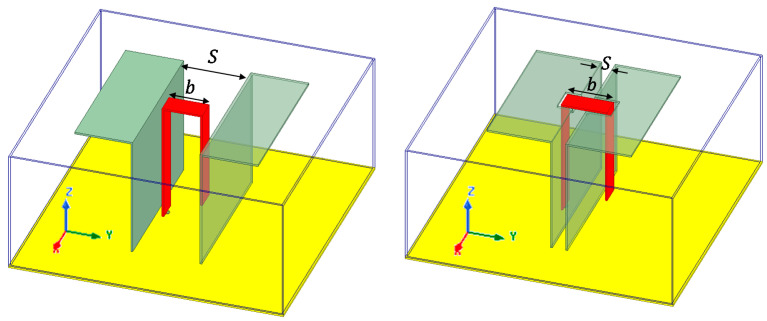
Cavity-backed conventional ME dipole antenna (**left**) and original proposed structure (**right**).

This paper shows the benefits of a new positioning of the Γ-shaped excitation probe compared to a conventional ME dipole antenna in terms of impedance wideband behavior. Illustrations of these two configurations are given in Figure 1. The magnetic dipole is constituted from the two vertical plates of the L-shapes, whereas the electric dipole is constituted of the two horizontal plates of these same shapes. The excitation is achieved by means of a Γ-shaped probe at the center of the antennas. For the conventional ME structure (Figure 1, left), this probe is located between the two arms of the magnetic dipole, and for the proposed solution (Figure 1, right), it is located at the outer of these two arms. The apertures on the electric dipoles to insert the probe can be noticed. The two structures exhibit the same external dimensions and are fulfilled with a dielectric material. A cavity of the same height as the ME dipole surrounds the structure. The objective is to design compact and low-profile antennas covering all the GNSS bands. Therefore, comparison of both structures is done using the same cavity size (given in the next section, see Table 1). Due to this constraint and the targeted bands (from 1.16 to 1.30 and from 1.56 to 1.61 GHz), structure 1 (conventional ME), providing only two resonances, presents a dual-band behavior as illustrated in Section 3. A wideband behavior with this structure starting from 1.16 GHz is not achievable without increasing the cavity height. Here, the decision was made to present the dual-band version of the classical ME antenna to better show the benefits of the proposed solution. In any case, all the GNSS bands could not be covered with structure 1 within the targeted external dimensions, whereas this objective is achieved with the proposed structure 2, as demonstrated in the following.

This idea to place the probe outer from the magnetic dipole comes from the structure in [7] proposed by J. Zeng and K.-M Luk in 2018, where the magnetic dipole was opened, creating a defected ground structure resulting in a wider BW; with the Γ-shaped probe located at the same position as the classical ME dipole. Here, openings in the electric dipole are created to insert the probe outer from the magnetic dipole and to excite the structure; this operation modifies the working process of the antenna. This will be developed in Section 3 of the paper. This specific placement of the probe creates a new controlled resonance for the structure, which enhanced the total BW of the antenna. A comparison of the conventional cavity-backed ME dipole (structure 1) and the original proposed structure (structure 2) with same external dimensions is proposed in this paper.

## 3. Antenna Design and Analysis

### 3.1. Geometry and Comparison with Conventional ME Antenna

**Figure 2:**
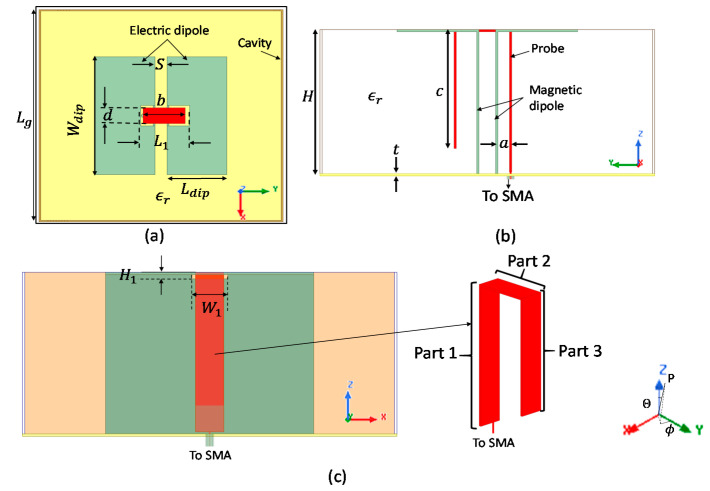
Structure of the proposed antenna, top view (**a**), side view of the structure (**b**), and 3D view of the probe (**c**).

The geometry of the considered antenna is given in Figure 2. As explained in the previous section, it consists of a ME dipole surrounded by a copper cavity and fed by a Γ-shaped probe (in red in Figure 1 and Figure 2). The cavity wall height is as large as the dipole height. The Γ-shaped probe presents a horizontal part 2 of length ‘b’. If this parameter ‘b’ is smaller than the interspace ‘S’ between the vertical plates, the structure is similar to the conventional ME one [1] (structure 1, Figure 1, left). If ‘b’ is larger than ‘S’, then the vertical parts 1 and 3 of the probe are located outside of the vertical plates, and the horizontal part 2 goes through apertures of dimensions $W_{1}$ × $L_{1}$ and $W_{1}$ × $H_{1}$ in electric and magnetic dipoles, respectively (structure 2, Figure 1, right). This original configuration results in a wider BW, as explained in the following. In both cases, the cavity is filled with polypropylene (PP) dielectric substrate ($\epsilon_{r}=2.26$, tanδ=0.0026 [18]) to ensure the mechanical robustness of the antenna (strong accelerations) and reduce side dimensions. The total size of the proposed antenna is 0.34 × 0.34 × 0.15$\lambda_{L}^{3}$ (90 × 90 × 40 mm^3^), where $\lambda_{L}$ is the wavelength at the lowest frequency (1.14 GHz).

All the simulations are performed using Ansys HFSS software [19]. To illustrate the benefits of placing the probe outside of the vertical walls, the two structures 1 (*b* < *S*) and 2 (*b* > *S*), of same external dimensions $L_{g}=90\;\mathrm{mm}$ and *H* = 40 mm are tuned and compared. All the dimensions after dichotomous optimization are summarized in Table 1. The reference point for parameter ‘*a*’ is the internal edge of closest vertical plate.

**Figure 3:**
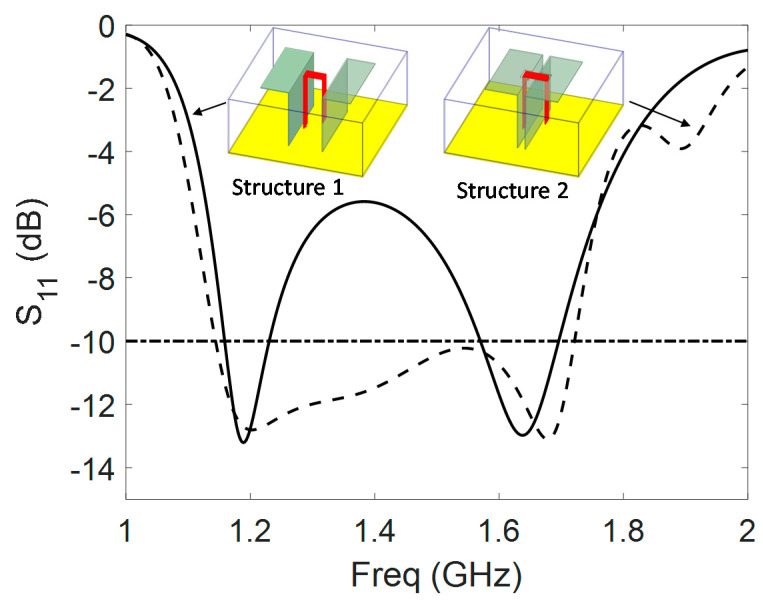
Comparison of reflection coefficient $S_{11}$ for structures 1 and 2.

Structure 1 is tuned to cover as many GNSS bands as possible, but as it can be seen from Figure 3, the objective to cover all the GNSS bands from 1.16 to 1.61 GHz is not achieved. Structure 1 presents only two resonances respectively on the vertical plates (magnetic dipole) and on the horizontal plates (electric dipole). Structure 2 was also tuned and presents a wide BW from 1.14 to 1.72 GHz covering all the GNSS bands. The wideband behavior of structure 2 is induced by the presence of a third resonance attributed to the probe itself, as explained in the next section. In addition, polypropylene substrate enables a size reduction of 33% for the ground plane area of structure 2 compared to the same structure in air $\left(\left(b>S\right),\;\epsilon_{r}=1\right)$.

### 3.2. Analysis of Structure 2

**Figure 4:**
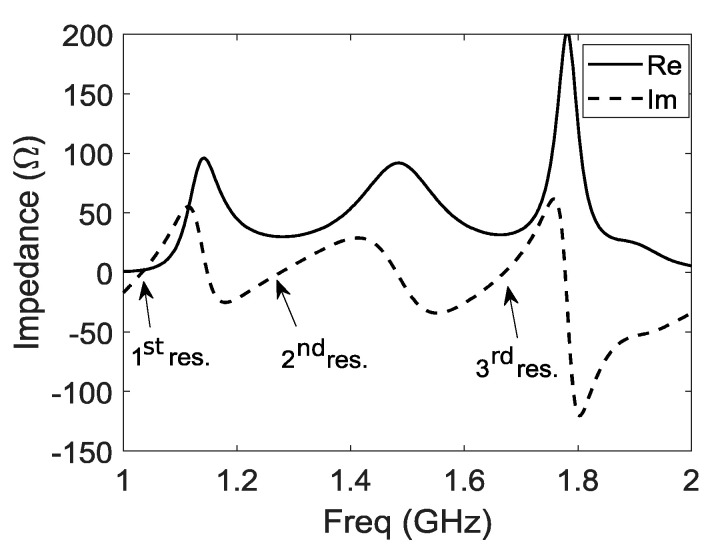
Input impedance $Z_{11}$ of structure 2 versus frequency, real part in continuous line, and imaginary part in dashed line.

The input impedance of structure 2 is shown in Figure 4. The first resonance comes from the horizontal part of the antenna and the cavity. Around 1.16 GHz, the antenna acts like a folded monopole antenna. Edges of the horizontal plates and cavity resonate respectively in λ/4 and  λ/2. The second resonance around 1.27 GHz corresponds to the magnetic component, due to vertical plates with λ/4 length. The third resonance (around 1.67 GHz) comes from the specific position of the probe. Parts 1 and 2 (Figure 2c) act as a microstrip line and excite the ME dipole by coupling. Part 3 acts as an open λ/4 stub (capacitive) and enables the high band adaptation by adjusting its length ‘c’. This position of the probe enables the miniaturization of the cavity by counteracting the inductive part induced by the small cavity and makes the wideband behavior of the proposed structure 2.

**Figure 5:**
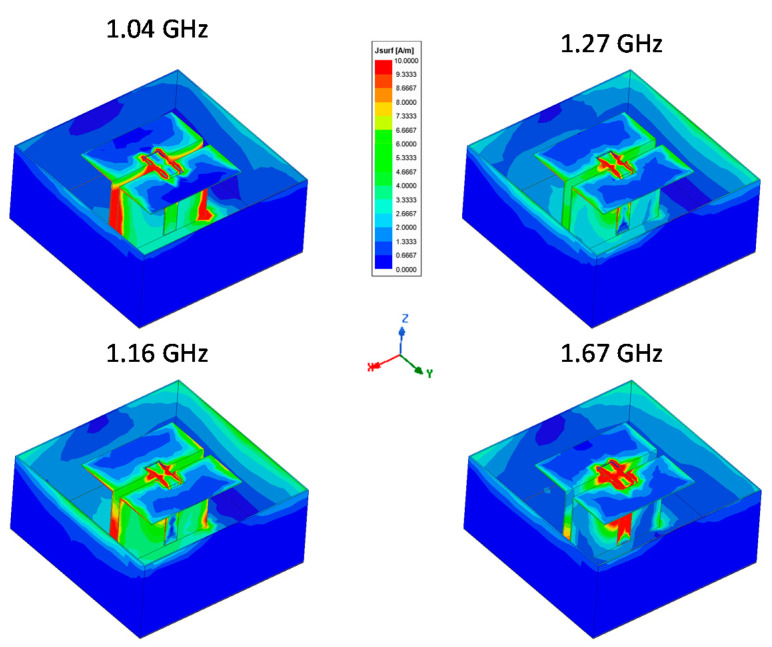
Current distribution on the antenna at resonance frequencies.

To understand the working process of the antenna, the current distribution of the antenna for resonance frequencies is shown in Figure 5.

This technique, also used in microwave engineering (e.g., [20]), allows the identification of strong currents on the structure for specific frequencies and helps with comprehension of working mechanisms involved. At 1.04 GHz, the dominant current is at the edge of the horizontal plates and rotates; this behavior is also observed at 1.16 GHz, where the horizontal and vertical parts are near their resonance frequencies. At 1.27 GHz, we can observe the strongest currents on the vertical part of the magneto-electric structure. The current distribution corresponds to a quarter wavelength patch distribution with a close loop current thanks to the antenna probe. At 1.67 GHz, the strongest current is located on the part 3 of the probe, corresponding to a λ/4 stub. For all the operating band, we can notice the current on the cavity, which is mainly concentrated on walls parallel to the magneto-electric structure. The cavity enables a steady gain for the antenna. 

### 3.3. Numerical Radiation Characteristics of Structure 2

**Figure 6:**
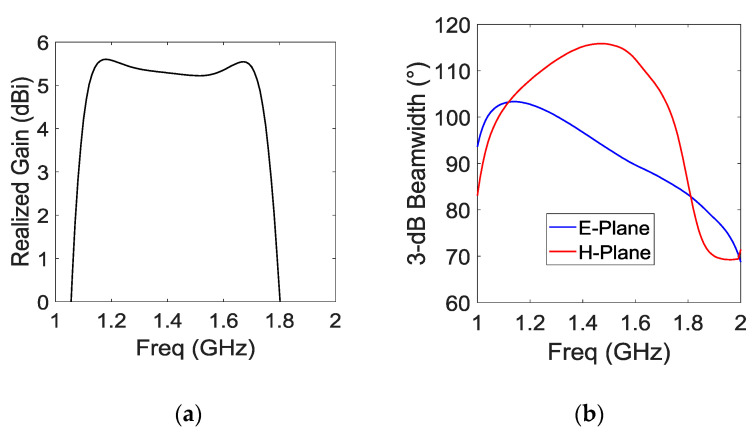
Input realized broadside gain (**a**) and 3 dB beamwidth (**b**).

**Figure 12:**
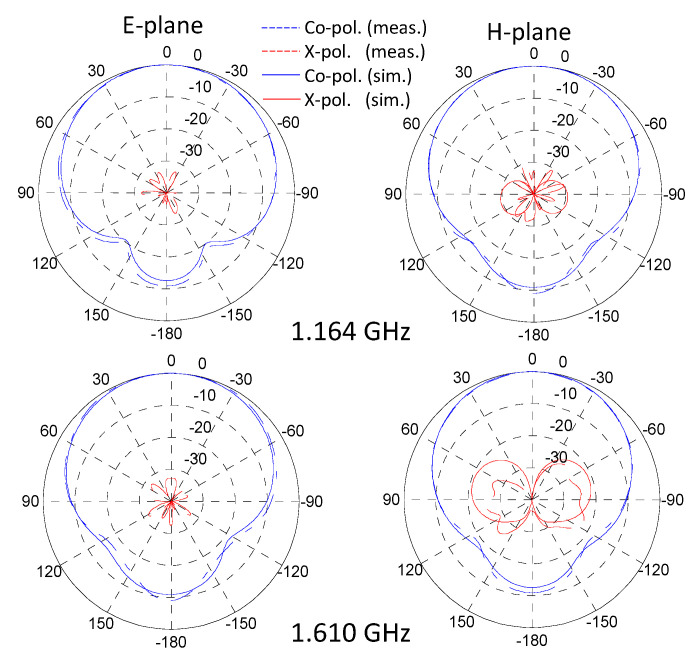
Simulated and measured normalized radiation pattern in E and H planes at extrema GNSS frequencies.

The cavity and the substrate enable very steady radiation properties for the antenna, which is important in the case of GNSS applications. Figure 6a shows the broadside realized gain versus the frequency. The gain bandwidth is 38.1% from 1.125 GHz to 1.655 GHz with a mean value of  5.1 dBi. The low gain variation (<0.5 dBi) is obtained thanks to substrate cavity filling. This antenna is also widebeam with a stable 3 dB beamwidth ≥90° in all the GNSS band for E and H planes (YoZ and XoZ planes respectively, Figure 6b). Normalized radiation patterns at 1.164 and 1.610 GHz are presented in Figure 12.

### 3.4. Parametric Analysis

**Figure 7:**
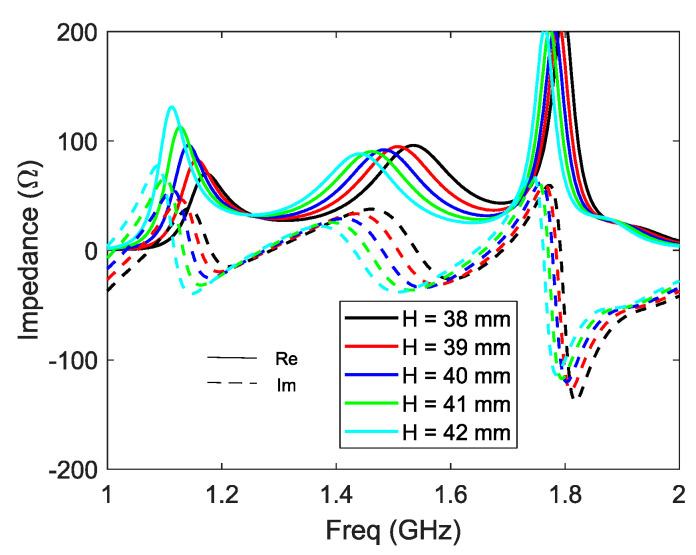
$Z_{11}$ vs. H.

A parametric analysis of structure 2 is performed to evaluate the sensitivity of the design and before all as design rules of such structures. This study is performed by starting from the tuned antenna and modifying the parameters one by one. The variation of impedance induced by the modification of the cavity height ‘*H*’ is shown in Figure 7. This parameter has a strong impact on the amplitude and the frequency of the first resonance and on the frequency of the second resonance. Decreasing ‘*H*‘ induces a higher and weaker first resonance and moves higher in the second frequency. This observation is in agreement with the origin of the resonances shown above (Figure 4).

**Figure 8:**
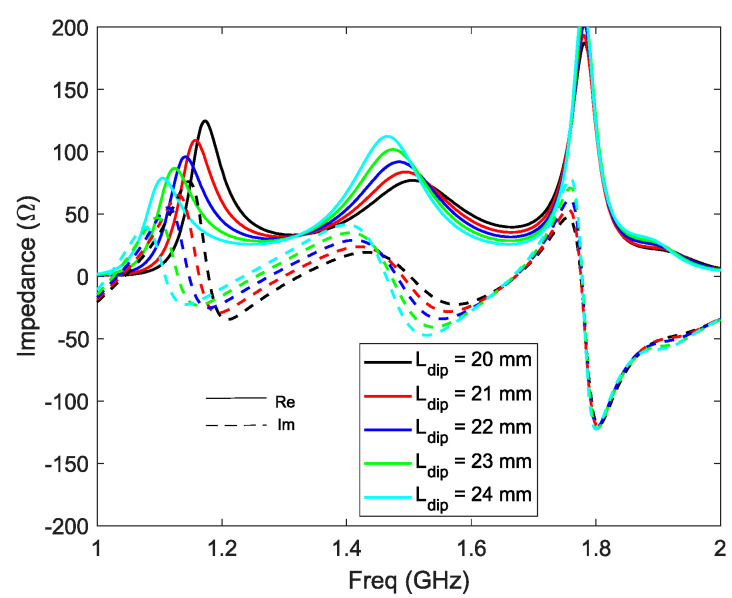
$Z_{11}$ versus $L_{dip}.$

Modification of the length of the horizontal plate ‘$L_{dip}$’ parameter has mainly an impact on the first resonance of the antenna (Figure 8). A longer horizontal plate will lead to a lower (in frequency) and weaker (in amplitude) first resonance; it will also have a lesser impact on the second resonance by increasing the capacitive part due to the larger surface of the horizontal plates over the ground plane.

**Figure 9:**
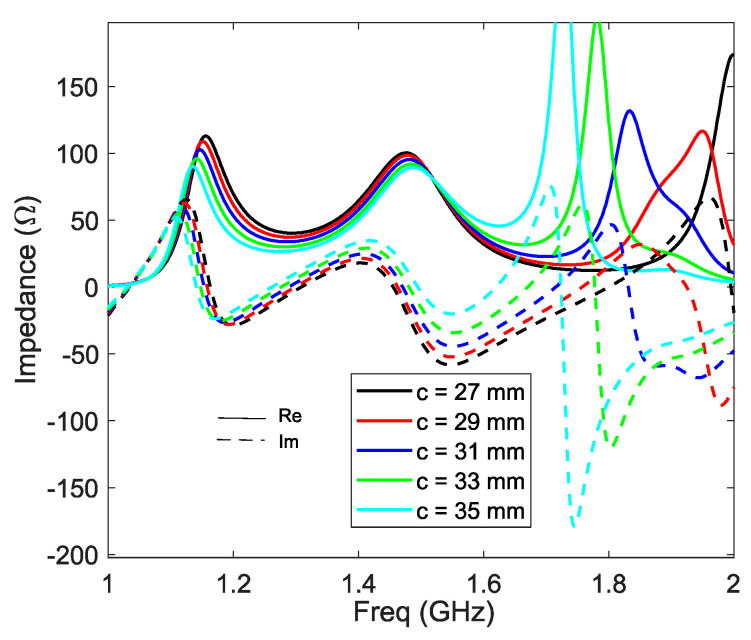
$Z_{11}$ vs. c.

The evolution of the input impedance versus the length of the vertical probe part ‘*c*’ is visible in Figure 9. As expected, the variation of ‘*c*‘ has mainly an impact on the third resonance of the antenna, shifting it higher by increasing its length. 

Some parameters such as ‘*a*’, ‘$W_{dip}$’, ‘*S*’, or ‘*d*’ are used to adjust the coupling between the three resonances and optimize the input impedance. For example, the variation of impedance induced by the modification of the space between the probe and the vertical plate ‘*a*’ impacts strongly the imaginary part of the input impedance, making it more capacitive when larger. This can be explained because part 1 of the probe (cf. Figure 2) is a transmission line over a ground plane, and the distance between these two elements modifies the value of the induced inductance. At last, ‘$L_{g}$’ is important for the lower band matching of the antenna. A summary of the influence of main dimensions on the behavior of the antenna is proposed in Table 2 as a guideline to design such an antenna.

## 4. Experimental Results

**Figure 10:**
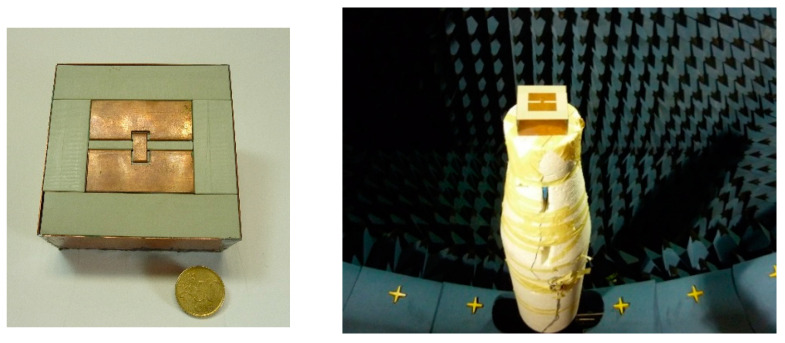
Photograph of the manufactured antenna (**left**) and radiating measurements (**right**).

**Figure 11:**
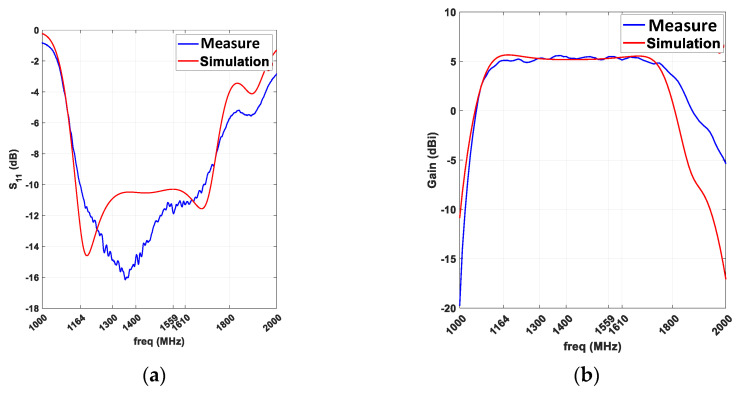
Simulated and measured $S_{11}$ (**a**) and broadside realized gain (**b**).

To validate the numerical results, a prototype, shown in Figure 10, is manufactured and measured. For scattering parameters measurements, Agilent E8363A network analyzer was used. The measured $S_{11}$ compared with the simulated one is shown in Figure 11. As can be seen, the measured −10 dB BW is 1.16–1.7 GHz. The small difference is attributed to manufacturing tolerances on prototype fabrication. For radiation measurement, a Satimo SG 24 near field anechoic chamber was used (Figure 10). The measured gain is close to the simulated one; it is stable all over the BW at approximately  5.1 dBi (Figure 11), and the measured radiation efficiency is over 84% for all the GNSS bands.

The simulated and measured normalized radiation patterns in E and H planes for extrema frequencies of GNSS bands are visible in Figure 12. A slightly higher but still negligible cross-polarization can be observed at 1.61 GHz but a good agreement is found. A comparison of the main simulated and measured radiation characteristics is shown in Table 3. X-pol column corresponds to the rejection level of the cross-polarization compared to co-polarization. The given values are the worst case ones over the GNSS bands. A high FTBR is observed due to the cavity with very low cross-polarization levels. Finally, a good agreement between simulation and measurements is found for this antenna.

A comparison of this structure with other linearly polarized cavity antennas is proposed in Table 4. From this table, we can see that the proposed antenna exhibits a small size compared to other linearly polarized magneto-electric cavity-backed antennas with relatively large BW. We can also notice that the cavity height of [6] is smaller than the radiating element size. This table also shows that the proposed antenna exhibits the lowest maximum cross-polarization level and the highest HPBW. This observation is important considering the targeted application, which requires covering a large angular range to get signals from the satellites. The authors are aware that the presented structures for comparison were published a few years ago. However, to their knowledge, no more recent structures match the requirements of the targeted application (cavity-backed and wideband antenna). 

## 5. Conclusions

A small size cavity-backed ME dipole antenna of global size 0.34 × 0.34 × 0.15λ^3^_L_ (90 × 90 × 40 mm^3^) is presented in this paper. Size reduction is achieved using polypropylene substrate to fill the cavity. In comparison to conventional ME antenna, the Γ-shaped probe is located outer from the magnetic dipole; an impedance BW ($S_{11}<-10\;\mathrm{dB}$) of 40.5% is achieved by the excitation of an additional resonance compared to the original ME dipole antenna, which is due to the positioning of the probe outside of the vertical plates. This makes it possible to achieve a wider BW and enables the antenna to cover all of the GNSS bands. The radiation properties are found to be very stable with frequency for the broadside gain as well as for the radiation pattern in the E and H planes. Low cross-polarization levels and a 3 dB beamwidth of over 89° for both planes are measured. A prototype was manufactured, and a good agreement is found between simulation and measurements. Typical applications of such compact antenna are UAVs and flying vehicles at medium and high altitude.

A combination of this specific probe placement with parasitic elements for linearly and circularly polarized magneto-electric antennas will be the subject of future works.

## Figures and Tables

**Table 1:** Dimensions of the two considered structures.

Structures	1		2	Structures	1		2
Parameters	Value (mm)			Parameters	Value (mm)		
$L_{g}$	90			a	3	−4	
H	40			b	12	15.5	
$L_{dip}$	19	22		c	33		
$W_{dip}$	50			d	6.75		
S	22	4.5		t	0.6		
$W_{1}$	-	8.75		$L_{1}$	-	17.5	
$H_{1}$	-	1.6		-	-	-	

**Table 2:** Parametric study synthesis.

Param.	Var.	1st res.		2nd res.		3rd res.	
-	-	*Freq.*	*Ampl*	*Freq.*	*Ampl*	*Freq.*	*Ampl*
S	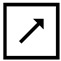	−−	+	+	+	+	−−
H	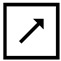	−−	++	−−	=	=	=
$L_{g}$	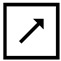	=	−−	−−	−	−	+
$L_{dip}$	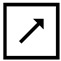	−−	−−	=	++	=	++
*a*	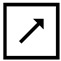	=	++	=	−−	=	=
*b*	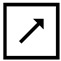	=	−−	=	++	−−	−−
*c*	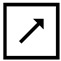	=	=	=	=	−−	−

**Table 3:** Comparison of main radiation characteristics (from 1.16 to 1.61 GHz).

	3 dB Beamwidth	FTBR	X-Pol	Real. Gain
Sim.	92°	>10.9 dBi	54 dB	5.2
Meas.	89°	>9.1 dBi	32.8 dB	5.1

**Table 4:** Comparison of the proposed antenna with state of the art.

Reference.	Dimensions (λ_c_)	Volume Compared to [4]	Impedance Bandwidth	Minimum HPBW	Max X-Pol Level
4	0.93 × 1.22 × 0.36	100%	88%	55°	−20 dB
13	0.967 × 0.967 × 0.173	39.6%	54.8%	30°	−20 dB
14	1.6 × 1.18 × 0.34	157.2%	68.8%	60°	−30 dB
15	0.86 × 0.76 × 0.23	36.8%	76%	60°	n/a
This work	0.41 × 0.41 × 0.18	7.4%	40.5%	89°	−32.8 dB

## Data Availability

Not applicable.
